# Vertical skeletal changes after extraction and non-extraction treatment in matched class I patients identified by a discriminant analysis: cephalometric appraisal and Procrustes superimposition

**DOI:** 10.1186/s40510-017-0198-5

**Published:** 2017-12-18

**Authors:** Philipp Beit, Dimitrios Konstantonis, Alexandros Papagiannis, Theodore Eliades

**Affiliations:** 1 Clinic of Orthodontics and Pediatric Dentistry, Center of Dental Medicine, Faculty of Medicine, University of Zurich, Zürich, Switzerland; 2 Department of Orthodontics, School of Dentistry, National and Kapodistrian University of Athens, Athens, Greece

**Keywords:** Extractions, Vertical dimension, Class I, Discriminant analysis, Treatment time

## Abstract

**Background:**

In the long-lasting debate of extraction versus non-extraction treatment, the impact of extractions on the skeletal vertical dimension remains rather unclear. The aim of this retrospective research study was to obtain a bias-free sample of morphologically similar borderline patients treated with or without extraction of the four first premolars and to retrospectively evaluate the vertical changes that occurred.

**Methods:**

A borderline sample of 83 patients, 41 treated with four first premolar extractions and 42 treated without, was obtained by means of discriminant analysis applied to a previously investigated parent sample of 542 class I patients. The pretreatment and posttreatment cephalometric radiographs were analyzed digitally, and seven measurements were assessed for vertical skeletal changes. Also, average tracings between the two treatment groups were evaluated using the Procrustes superimposition method.

**Results:**

The variables of SN to Go-Gn and *Y*-axis showed adjusted intergroup differences of − 0.91° and − 1.11° (*P* = 0.04). Comparing the mean intra-group differences of all the variables simultaneously, a significant difference was found between the two treatment groups (overall *P* value = 0.04). In the extraction group, only the gonial angle showed a significant decrease (*P* = 0.01) while the overall *P* value evaluating the intra-group differences between pre- and posttreatment was significant (overall *P* value < 0.01). In the non-extraction group, the variable of N-ANS/N-Me showed a significant decrease (*P* = 0.02) and the overall *P* value evaluating the intra-group differences between pre- and posttreatment was also significant (overall *P* value < 0.01). Differences in treatment duration were assessed using a log-normal model and showed that extraction treatment lasted significantly longer than non-extraction treatment (*P* < 0.01).

**Conclusions:**

The borderline group of patients identified by the discriminant analysis exhibited similar morphological characteristics at treatment’s onset; therefore, the posttreatment changes could safely be attributed to the choice of extraction or non-extraction treatment and not to pre-existing differences. Treatment choice had an impact on the patients’ vertical skeletal dimensions. Patients treated with four first premolar extractions showed a slight decrease in the vertical skeletal measurements, whereas non-extraction patient treatment showed a slight increase. The treatment time was also significantly higher in the extraction group.

## Background

Extractions are routinely implemented in orthodontics mainly to address crowding and reduce dentoalveolar protrusion. While the impact of extraction and non-extraction treatment on the soft tissue response, smile esthetics, stability, and other parameters has been extensively investigated [1–3], the literature is rather inconclusive with regard to the impact of the extractions on the vertical dimension.

Control of the vertical dimension during orthodontic treatment is challenging [4]. Still, the open bite manifestations can range from a variety of skeletal, dental, and functional features including increased mandibular plane and/or gonial angle, altered anterior or posterior facial heights, weak orofacial musculature, inadequate lip seal, and anterior tongue position or thrust [5, 6]. Different treatment techniques or extraction patterns have been suggested to address the aforementioned discrepancies [7–9]. Often, orthodontists tend to extract in patients with increased anterior facial height. According to the wedge hypothesis, the extraction of four premolars or molars and the subsequent protraction of the posterior teeth lead to a counterclockwise rotation of the mandible, thus maintaining or increasing the overbite [8, 10, 11]. Although this theory is quite popular, it is not evidence-based according to relative investigations [7]. Contradictory reports in the literature fail to reach a consensus whether or not extractions have a definite effect on the vertical dimension [12–20].

On a research note, often in the literature, the matching process in retrospective surveys is rather inadequate since the compared groups are not morphologically similar [8, 15, 21, 22]. The improper matching of the groups inevitably introduces susceptibility bias, which is defined as the difference in prognostic expectations due to pre-existing differences between and/or among treatment groups. When contemplating between different-mutually exclusive-treatment approaches or techniques, a clinician takes into careful consideration the patient’s morphological features. These features usually include cephalometric and model measurements along with other parameters like patient’s age and sex. In statistics, such patient’s features that lead a clinician to a specific treatment decision are called confounding variables.

Discriminant analysis is a statistical multivariate technique that deals simultaneously with a large number of confounding variables. In contemporary orthodontic research, discriminant analysis has been used in order to identify homogenous samples that cannot be discriminated with regard to a specific treatment modality. The homogeneity of the groups regarding the variables included in the discriminant analysis can ensure that all patients are borderline and equally susceptible to alternative treatments. As shown in the literature, borderline samples are ideal for various posttreatment comparisons [1, 23–25].

It was therefore the aim of the present retrospective research study to evaluate a bias-free sample of borderline patients treated with or without four first premolar extractions and to assess the vertical skeletal changes occurred.

## Methods

To overcome common methodological errors seen in orthodontic literature and to eliminate susceptibility bias, it was decided to obtain a borderline sample in regard to extractions that derived from a large parent sample of class I patients of a previous investigation [23]. The parent sample consisted of 542 randomly selected subjects, treated at the graduate Orthodontic Clinic of the School of Dentistry of the National and Kapodistrian University of Athens, Greece and in five different private orthodontic offices in Athens, Greece. The decision to select patients especially from a university clinic where patients were treated by a numerous residents and clinical instructors and from different private clinical settings was made in order to eliminate the possibility of selection and proficiency bias.

All patients were Caucasian male or female with a class I dental and skeletal malocclusion, no transverse discrepancies, and a full complement of teeth excluding the third molars. They had no history of clefts or any other dentofacial deformities, and they never had received any previous orthodontic treatment or orthognathic surgery. Still, the patients included did not present with extensively decayed teeth that could influence the clinician towards extraction, and according to the charts, when extractions were decided, it was solely for orthodontic purposes. Out of the 542 patients, of which 331 were female and 211 male, 427 were treated by non-extraction and 153 with extraction of the four first premolars. The parent sample was collected in 2013, while the identification of the borderline sample for the present investigation took place in 2017.

All patients received orthodontic treatment with preadjusted edgewise appliances in both arches and had a complete set of diagnostic records including initial and final lateral cephalometric and panoramic radiographs along with dental casts and detailed treatment charts. Neither extra- nor intra-oral appliances or temporary anchorage devices were used during treatment. However, in regard to treatment mechanics in the extraction cases and according to patients’ charts, after crowding was addressed by the retraction of the anterior teeth, the implemented biomechanics aimed at closure of the remaining spaces by protraction of the posterior teeth. All cephalograms were taken in the natural head position and were traced and analyzed using ViewBox 4.0.1.7. The research protocol was approved by the Ethics and Research Committee of the National and Kapodistrian University of Athens, Greece (ref. 311/21.09.2016).

The parent sample was then subjected to a stepwise discriminant analysis, which included all variables that could possibly influence a clinician’s decision towards extraction treatment. These variables were 26 cephalometric measurements, six dental cast measurements, and the demographic variables of age and sex [23]. Hence, a reliable representation of all of the patient’s dental, skeletal, and soft tissue traits that could possibly swing the pendulum towards one of the two possible treatment modalities was achieved.

Patients were predicted to belong to the extraction or the non-extraction group according to their discriminant score. The discriminant score for each patient was the sum of the multiplication of the discriminating variables with their standardized canonical discriminant function coefficients. Subsequently, the discriminant score can be considered a weighted linear combination (sum) of the discriminating variables.

**Fig. 1:**
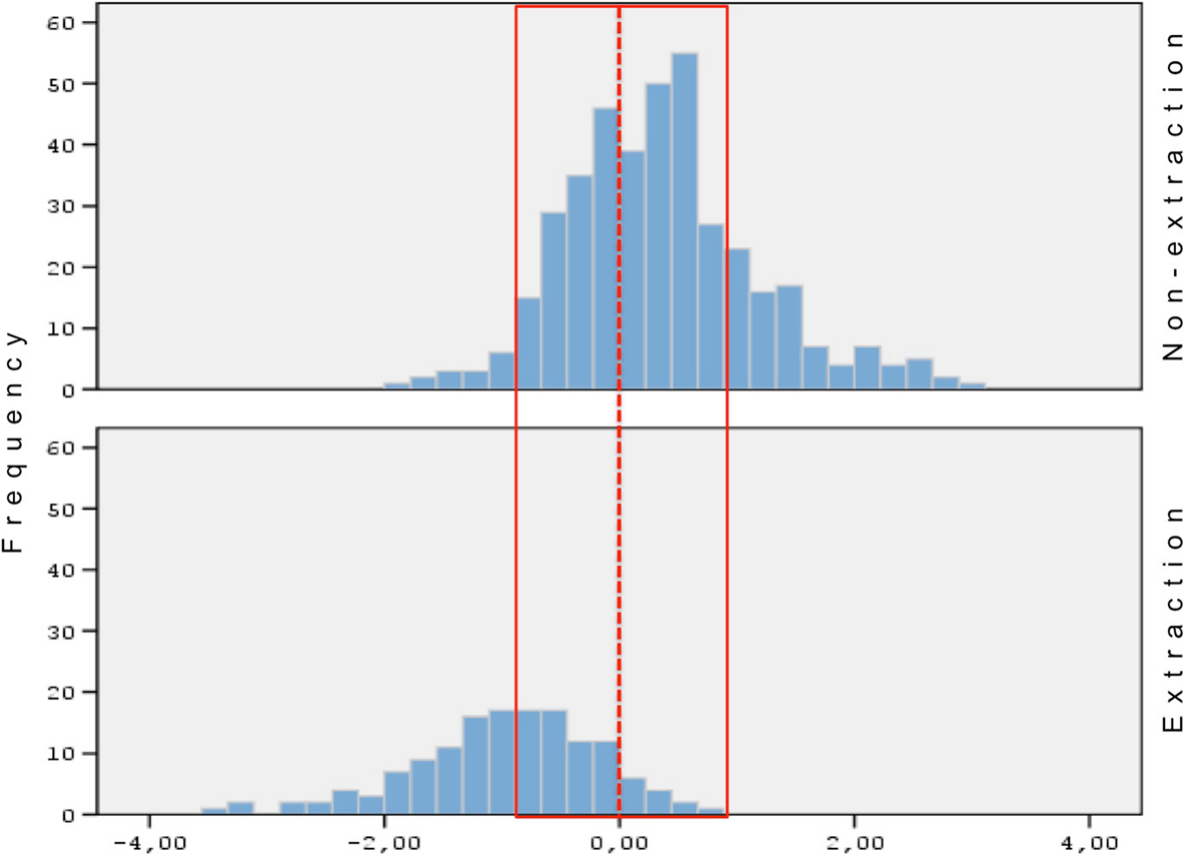
Histogram of Fisher’s discriminant scores distribution for the extraction and the non-extraction groups of patients. The red vertical line indicates the optimal cutoff point at 0, and the red dashed vertical lines indicate the range within 1 standard deviation (0.94) that the borderline sample was selected

According to the discriminant analysis, each patient was assigned a discriminant score that ranged from − 3.48 to + 3.07. Patients that received a negative score were predicted to be treated with four premolar extractions, whereas patients receiving a positive score were more likely to be treated by non-extraction (Fig. 1). The further away a patient’s score was drawn from 0 (the cutoff point), the more definite the treatment decision was, thus classifying the patient to either the “clear-cut” extraction or non-extraction group. Conversely, patients with discriminant scores around 0 exhibited a significant degree of morphological similarity and therefore could not be clearly classified to either one of the two groups.

**Fig. 2:**
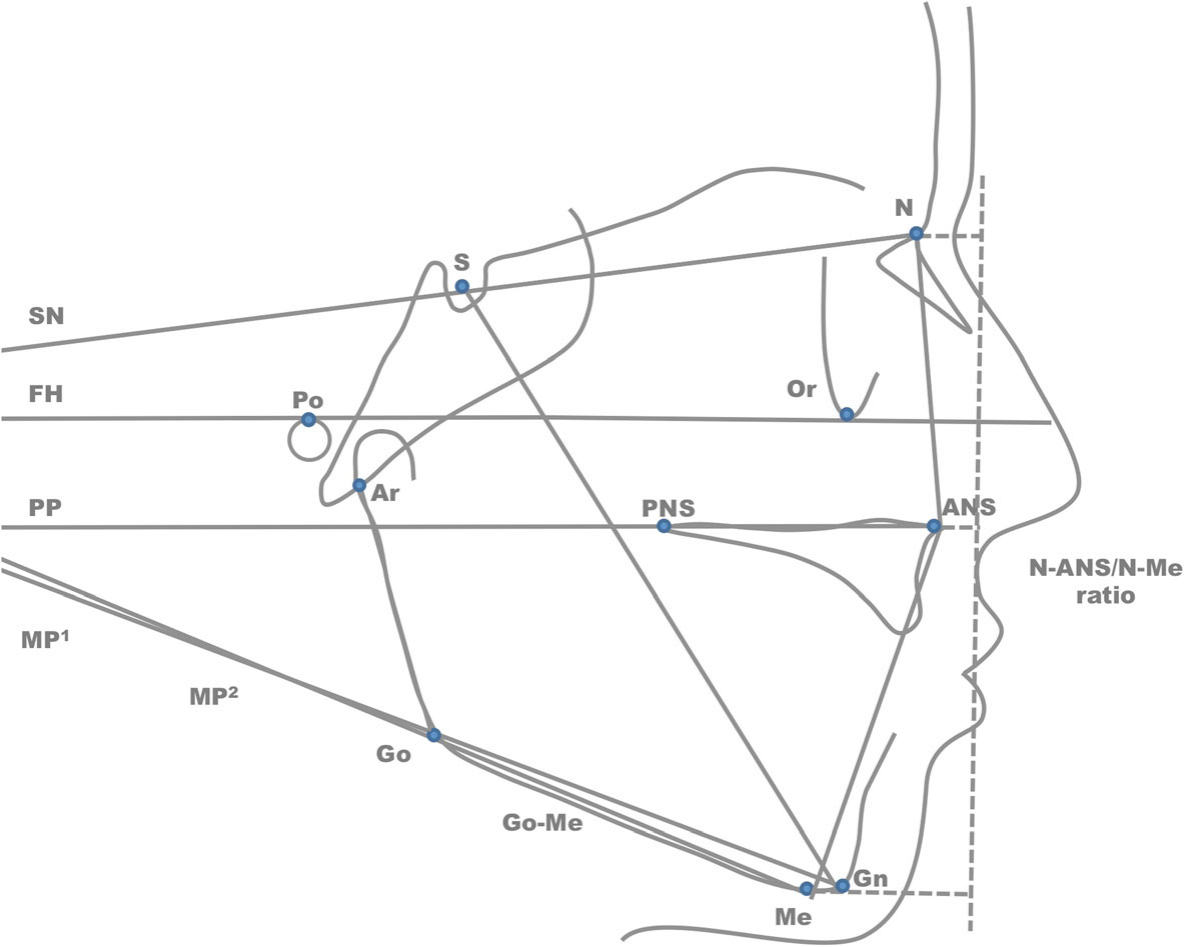
Points, planes, and lines used in the cephalometric analysis. Points: sella (S), nasion (N), po (Porion), orbitale (O), articulare (Ar), anterior nasal spine (ANS), posterior nasal spine (PNS), gonion (Go), menton (Me), gnathion (Gn). Planes: sella-nasion (SN), Frankfurt horizontal (FH), palatal plane (PP), mandibular plane^1^ (Go-Gn), mandibular plane^2^ (Go-Me). Lines: sella to gnathion (S-Gn), dashed line perpendicular to FH

To assess the impact of the two different treatment approaches, seven commonly used variables that measure the skeletal vertical dimension were employed. Of the seven variables, six describe angles and are shown in degrees and one variable is a ratio (N-ANS/N-Me). The orientation of the mandibular plane to the anterior cranial base was described by the following angles: FMA (Frankfurt horizontal (FH) plane to mandibular plane (MP) derived by the line connecting the landmarks gonion and menton); SN to Go-Gn angle that is formed at the intersection of the lines passing from the landmarks Sella to nasion and gonion to gnathion; and *Y*-axis which is the angle formed at the intersection of the line Sella to Gnathion to Frankfurt horizontal plane. Also, the gonial angle formed by the points menton-gonion-articulare was assessed. The anterior facial height was further evaluated by the variable of N-ANS/ANS-Me derived by the ratio of the projections of both measurements to the perpendicular on FH plane. Additionally, the cant of the palatal plane (PP) in relation to the cranial base and the mandibular plane was evaluated utilizing the measurements of palatal plane to SN and to MP respectively. All cephalometric landmarks, planes, and lines used can be seen in Fig. 2.

Regarding the sample size calculation, we assumed 1.7° mean difference in FMA measurements between the two groups, with the standard deviation assumed to 2.75 in both groups. Setting the significance level at 5%, to achieve 80% power, 42 individuals were required in each group.

Descriptive statistics for the pretreatment variables for both groups, as well as descriptive and inferential statistics to assess the intra- and intergroup differences in vertical dimension changes, were performed. Since these variables were used in the previous discriminant analysis to identify the borderline cases, baseline differences between the two groups were quite unlikely. The mean differences from pretreatment to posttreatment measurements that occurred in each group were compared through regression models adjusting for baseline age and sex. Furthermore, paired *t* tests were calculated to assess the differences between the pretreatment and posttreatment measurements for the two groups separately. The joint significance of differences was measured using *F* tests. The significance level was predetermined at 5%.

All cephalometric tracings were performed by the principal investigator (XX). Additionally, all tracings were averaged and then superimposed using generalized Procrustes superimposition [26]. Procrustes superimposition was performed on the inter- and intragroup averaged hard tissue profile. Fifteen skeletal points were used as reference for the superimposition (A point, B point, sella, sheno-ethmoidale, nasion, orbitale, porion, basion, articulare, gonion, antegonial notch, menton, pogonion, anterior nasal spine, posterior nasal spine). More specifically, in our study, Procrustes superimposition takes two shapes, resizes them, and aligns them to minimize the sum of the squared distance between corresponding cephalometric points. This is a mathematically defined procedure, characterized by validity and repeatability of the results [27].

Additionally, evaluations were performed for both random and systematic errors of the method. To evaluate intra-examiner repeatability, with a table of random numbers, 20 subjects were selected—10 from each treatment group—and were retraced 3 weeks later by the same investigator. Also, to assess inter-examiner agreement, 20 subjects—10 non-extraction and 10 extraction—were randomly selected, and the principal investigator was evaluated against the second investigator (XX). The intra-class correlation coefficient (ICC) based on the variance components from a one-way analysis of variance was used. All statistical analyses were performed in STATA (version 13.0; Stata Corp, College Station, TX).

## Results

Finally, the borderline sample was comprised of 83 patients that were chosen around the optimal cutoff point (0) of the discriminant scores and within one standard deviation as shown in Fig. 1. Of the patients, 42 that were treated by non-extraction and 41 were treated with four first premolar extractions. Of the non-extraction patients, 24 were female and 18 were male; meanwhile, out of the extraction patients, 23 were female and 18 were male. The mean age for the extraction group was 13.71 years (SD 3.28) and for the non-extraction group 14.62 years (SD 3.84).

Descriptive statistics of the pretreatment variables for both extraction and non-extraction groups are listed in Table 1. As expected, the *P* values (*t* test for independent samples) showed no statistically significant differences between the two groups at treatment’s onset. This outcome was further confirmed by the *P* value for pretreatment differences in all outcomes (*P* = 0.59).

**Table 1:** Pretreatment differences between extraction and non-extraction groups with respect to demographic characteristics and cephalometric measurements

	Extraction	Non-extraction	
Variables	(*N* = 41)	(*N* = 42)	
*N*	Mean ± SD	Mean ± SD	*P* value
FMA (°)	28.37 ± 3.96	26.85 ± 5.86	0.17
N-ANS/N-Me	44.45 ± 1.82	44.58 ± 2.44	0.79
SN to Go-Gn (°)	34.04 ± 3.79	33.80 ± 6.37	0.83
*Y*-axis (°)	60.52 ± 3.88	59.46 ± 3.56	0.20
Gonial angle (Ar-Go-Me) (°)	129.00 ± 4.82	128.42 ± 6.46	0.65
Palatal plane to SN (°)	6.77 ± 2.51	7.42 ± 4.22	0.40
Palatal plane to MP (°)	27.72 ± 4.35	26.71 ± 6.85	0.42
Age	13.71 ± 3.28	14.62 ± 3.84	0.25
Males	18 (43.90%)	18 (42.86%)	0.92
Duration (years)	2.79 ± 1.16	1.8 ± 0.65	< 0.01
*P* value for overall difference in the cephalometric variables 0.59			

The intra-group differences between pre- and posttreatment measurements were also examined through paired *t* tests for the two groups separately. In the extraction group, all variables apart from the palatal plane to SN (mean difference, 0.38; 95% confidence interval (CI), − 033, 1.09; *P* = 0.29) showed a decrease, but statistical significance was found only for the gonial angle (mean difference, − 1.06; 95%CI, − 1.80, − 0.32; *P* = 0.01). Still, the overall *P* value evaluating the differences between pre- and posttreatment in the extraction patients was significant (overall *P* value < 0.01).

In the non-extraction group the variables of FMA, SN to Go-Gn, *Y*-axis and palatal plane to SN showed an increase whereas the variables of N-ANS/N-Me, gonial angle, and palatal plane to MP showed a decrease. However, of all these variables, only N-ANS/N-Me showed a statistically significant change (mean difference, − 0.52; 95%CI, − 0.97, − 0.08; *P* = 0.02). Yet, when all measurement differences were simultaneously evaluated in the non-extraction patients, the overall *P* value showed a significant change between pre- and posttreatment values (overall *P* value < 0.01). The results of the intra-group differences can be seen in Table 2.

**Table 2:** Comparisons of intra- and intergroup differences between the two treatment groups

	Extraction			Non-extraction					
Variables	Diff.^a^	95%CI	*P* value	Diff.^a^	95%CI	*P* value	Adjusted diff.^b^ 95%CI	*P* value	Overall *P* value^c^
FMA (°)	− 0.68	(− 1.56, 0.21)	0.13	0.37	(− 0.50, 1.25)	0.40	− 0.98 (− 2.20, 0.23)	0.11	0.04
N-ANS/N-Me	− 0.38	(− 0.83, 0.07)	0.10	− 0.52	(− 0.97, − 0.08)	0.02	0.16 (− 0.46, 0.78)	0.61	
SN to Go-Gn (°)	− 0.57	(− 1.19, 0.05)	0.07	0.32	(− 0.30, 0.93)	0.30	− 0.91 (− 1.77, − 0.06)	0.04	
*Y*-axis (°)	− 0.39	(− 1.18, 0.40)	0.33	0.70	(− 0.07, 1.48)	0.08	− 1.11 (− 2.19, − 0.03)	0.04	
Gonial angle (Ar-Go-Me) (°)	− 1.06	(− 1.80, − 0.32)	0.01	− 0.54	(− 1.27, 0.19)	0.15	− 0.52 (− 1.54, 0.49)	0.32	
Palatal plane to SN (°)	0.38	(− 0.33, 1.09)	0.29	0.45	(− 0.25, 1.15)	0.21	− 0.15 (− 1.12, 0.81)	0.76	
Palatal plane to MP (°)	− 0.70	(− 1.49, 0.08)	0.08	− 0.06	(− 0.84, 0.71)	0.87	− 0.60 (− 1.67, 0.47)	0.27	

*P* value evaluating the significance of all differences in the extraction group < 0.01; corresponding *P* value for the non-extraction group < 0.01

^a^Differences between post- and pretreatment measurements

^b^Adjusted for age and sex differences between the two groups

^c^Tests whether all differences between treatment groups equal zero by using an *F* test

Differences in treatment duration were assessed using a log-normal model, which showed that extraction treatment lasted significantly longer than non-extraction treatment (*P* < 0.01) (Table 1).

**Fig. 3:**
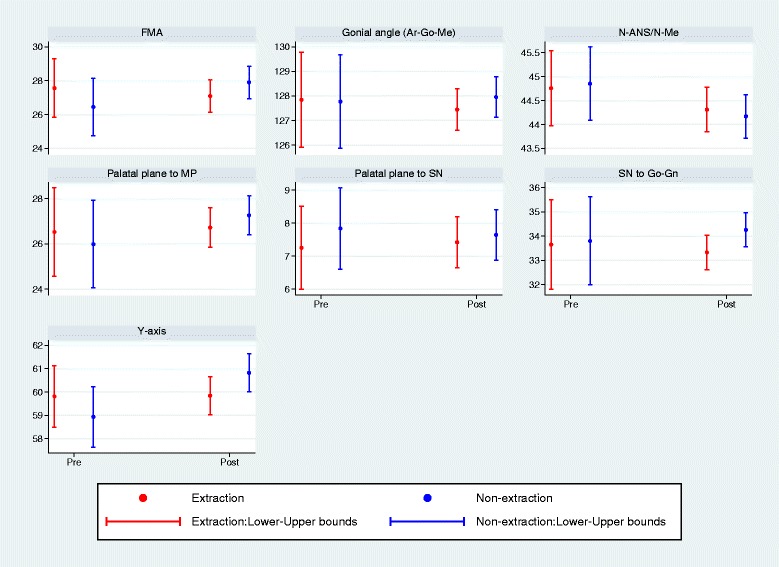
Comparisons between pre- and posttreatment measurements in the extraction and non-extraction group of patients

**Fig. 4:**
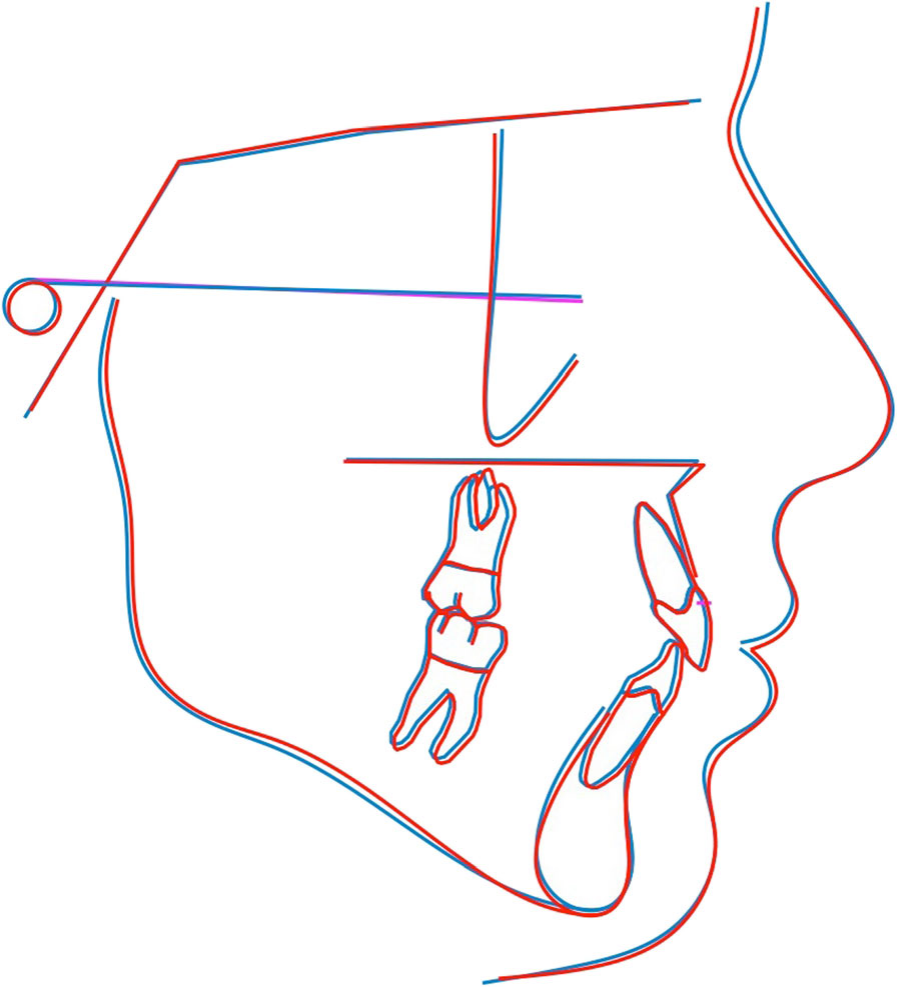
Average tracings at the start of treatment using Procrustes superimposition. Blue line, non-extraction; red line, extraction

**Fig. 5:**
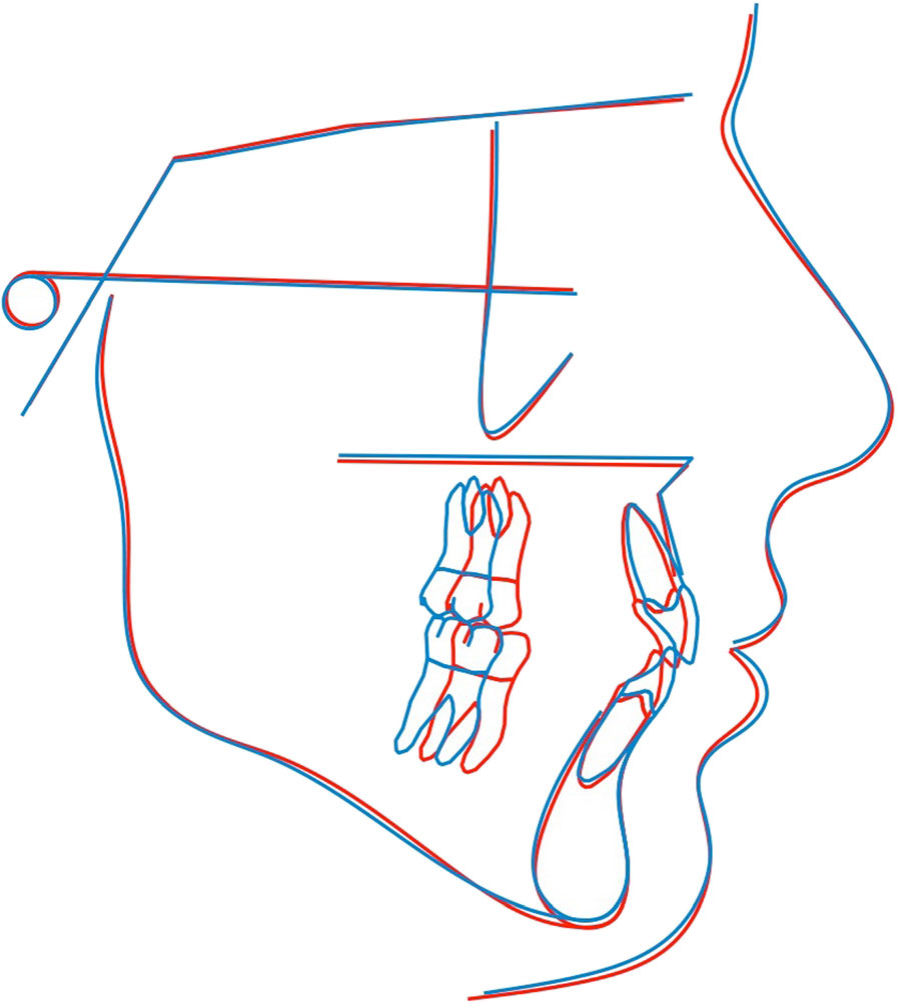
Average tracings at the end of treatment using Procrustes superimposition. Blue line, non-extraction; red line, extraction

When we compared the two groups, the differences of the mean change values for five out seven cephalometric variables were not statistically significant (Table 2). Contrariwise, the variables of SN to Go-Gn and the *Y*-axis showed adjusted differences of − 0.91 (95%CI, − 1.77, − 0.06; *P* = 0.04) and − 1.11 (95%CI, − 2.19, 0.03; *P* = 0.04) between the two groups. Considering the mean intra-group differences of all variables simultaneously, the *F* test suggested a statistically significant difference between the two treatment groups (overall *P* value = 0.04). The comparisons of the intra-group differences can also be seen in Table 2. Also, the intra-group trajectories of the extraction and non-extraction patients are depicted in Fig. 3. Average tracings and superimpositions between the two treatment groups can be seen in Figs. 4 and 5.

The results of the evaluations for random and systematic errors showed excellent agreement: ICC 0.99 (95%CI 0.99–1.00) for intra-examiner agreement and ICC 0.98 (95%CI 0.98–1.00) for inter-examiner agreement.

## Discussion

In this investigation, the use of the discriminant analysis ensured the morphological homogeneity of the extraction and non-extraction samples, thus eliminating the susceptibility bias commonly seen in orthodontic retrospective surveys [8, 15, 19, 28]. The borderline spectrum was comprised of patients with similar skeletal and dental features along with the vertical skeletal measurements. These statistically “unclassified” borderline patients with regard to extractions received extraction or non-extraction treatment that was decided by the orthodontist that they happened to visit. The effect of the orthodontic treatment on the vertical dimensions in the borderline spectrum of patients was then assessed through a cephalometric analysis which provided an objective assessment of the vertical skeletal changes that occurred. Due to the aforementioned methodology, the treatment outcomes can be safely attributed to the choice of treatment modality and to not to pre-existing dental or skeletal differences among patients.

Furthermore, Procrustes superimposition was conducted so that shape differences could be described. According to this mathematical model, in order to compare two configurations, we firstly adjust for size, and then align them, so that any effect of translation and rotation are removed. This method exhibits the advantage over the classical cephalometric approaches that all points are considered equally significant. Conventional superimposition planes, such as the Frankfurt horizontal (FH) plane, or the anterior cranial base plane (SN), although widely encountered in orthodontic literature, would imply that two points out of the whole would be of greater importance over the others. Additionally, by adjusting for size, pure shape differences could be observed, not affected by the scale factor.

As shown by the discriminant analysis applied to the parent sample of 542 class I patients, measurements that assess the vertical dimension like the *Y*-axis, FMA, ANS-Me did not show any discriminating power between the extraction and non-extraction group of patients. That occurred despite the fact that these measurements differed significantly between the two treatment groups [23]. Subsequently, with regard to the treatment decision, the vertical variation between patients was not taken into consideration by the clinicians upon treatment planning, despite the fact that the literature reports a possible vertical effect when a case is treated with extractions [18]. Still, the variable of lower crowding was the most important variable in deciding extractions as shown by the magnitude of its standardized canonical function coefficient (0.728) followed by the lower lip to E-plane (− 0.407), upper crowding (0.347), and overjet (− 0.219) [23].

Our results showed that in regard to the vertical skeletal measurements, the patients treated with extractions showed a slight decrease, which bordered at the traditional 5% level of statistical significance (*P* = 0.04) compared to their non-extraction counterparts. Despite the statistical significance though and because of the small-scale intergroup differences, it is open to discussion whether or not the results are clinically significant.

However, of the seven variables, only two (SN to Go-Gn and *Y*-axis) differed significantly between the two groups at the end of treatment, with them being decreased in the extraction group and increased in the non-extraction group of patients. Aras [12] examined open-bite cases and reported a significant decrease of the SN to Go-Gn angle after the extraction of the four second premolars and also after the extraction of the four molars but, in disagreement with our findings, reported a non-significant decrease after extraction of the four first premolars. Also, in contrast to our findings, Meral et al. [29] did not observe a significant intergroup posttreatment difference for the SN to Go-Me angle in their study. Furthermore, Kumari et al. [30] did not find any significant mean change difference for the *Y*-axis between the extraction and non-extraction cases. Moreover, the intergroup posttreatment differences for the *Y*-axis in Luppanapornlarp et al. [19] investigation can rather be attributed to the morphologically dissimilar pretreatment groups rather than to the treatment modality itself. In regard to the FMA angle, our results are in agreement with previous investigations that reported non-significant changes between the two treatment groups [17, 30]. Kocadereli et al. [15] also reported a slightly higher but not significant increase of the FMA and SN to Go-Gn angles in the non-extraction group of patients. Regarding the slight closure of the palatal plane to mandibular plane angle, our findings are in agreement with the findings of Kirschneck et al. [14].

Intergroup mean tracings were superimposed using Procrustes superimposition. The Procrustes method was chosen in order for all landmarks to be treated equally, and therefore, no points were arbitrarily considered to be of greater significance when compared to the others. Furthermore, all tracings superimposed were scaled to a standard size. As a result, pure shape changes could be detected, not affected by the influence of size [31]. In regard to the vertical dimension, the average tracings at the end of treatment showed a slight difference between the extraction and the non-extraction patients (Fig. 5). Still, the main changes were localized at the perioral area and were manifested as retraction of the anterior dentoalveolar units followed by subsequent retraction of the lips. In contrast to our results, Garlington et al. [18] observed in the cephalometric superimpositions a forward rotation of the mandible in 17 out of the 23 cases that were though treated with extractions of the four second premolars.

In the extraction group, almost all of the vertical measurements showed a non-significant decrease except for the gonial angle that was reduced significantly. Nevertheless, when all measurements were taken into consideration, the overall intra-group difference between pre- and posttreatment showed statistical significance. In regard to the FMA [8, 15, 19, 30] and the palatal plane to MP angle [14, 17, 29], our results are in agreement with those of other authors who also suggested a decrease after extraction treatment. Also, Kim et al. [8] reported a non-significant decrease of the palatal plane to MP angle after four first premolar extraction treatment in contrast to four second premolar extraction treatment that resulted in a significant increase of the aforementioned plane. Concerning the *Y*-axis, Kumari et al. [30] showed a significant intra-group increase for the extraction patients in contrast with our study where a slight non-significant decrease was found.

In regard to the gonial angle, a similar observation of a decrease but at a higher value (− 2.5, SD 4.5) was made by Kirschneck et al. [14] in an extraction group of patients. Other authors could not confirm any similar findings [8], while most did not assess changes in the gonial angle. Gonial angle though is an important parameter of the craniofacial complex giving an indication about the vertical parameters and symmetry of the facial skeleton. Any change in the gonial angle might be attributed to two distinctive causes. Either there is a true morphologic change in the angle between the ramus and the base of the mandible or there is a change in the location of the derived cephalometric landmark of articulare due to rotation of the mandible. Because of the curvature in the mandibular condyle, any minute change in the identification of articulare can have an impact on the gonial angle. Even though not statistically significant, most changes for vertical parameters were negative in the extraction group, thus showing a vertical decrease, which could lead to the alteration of the gonial angle.

In the non-extraction group, the angular measurements that assess the vertical skeletal changes in regard to the orientation of the mandible to the anterior cranial base showed a non-significant increase, thus being in accordance with the findings of other authors [14, 15, 17]. The statistical significance of the overall intra-group differences though shows that the treatment had a definite impact on the vertical skeletal dimension of the non-extraction patients by increasing it. In contrast to our study and other authors, Meral et al. [29] found a significant decrease in the palatal plane to MP angle (mean diff. − 1.5, *P* < 0.05) and in the SN to Go-Me angle (mean diff. − 1.5; *P* < 0.01) after a non-extraction therapy. Our results showed a decrease of − 0.52 for the anterior facial height ratio (N-ANS/N-Me), therefore differing from the reports of Kumari et al. [30] who observed a significant increase (mean diff. 1.1, *P* = 0.005) and of Sivakumar et al. [17] who also assessed a slight but not significant increase of 0.08 (*P* = 0.81) after non-extraction therapy.

The amount of initial crowding should also be taken into consideration when assessing the posttreatment vertical skeletal changes since these changes are closely related to the extent of tooth movement. In studies assessing the impact of extractions on the vertical dimension, the initial crowding ranges from slight to severe [8, 24, 28, 29], while in most studies, it is not reported [9, 12, 15, 17, 18, 30, 32]. Clinically, an orthodontist initially addresses the anterior crowding by moving the teeth into the extraction sites and then it is decided whether or not to close the remaining spaces from the posterior, the anterior, or reciprocally. In cases with severe tooth-arch discrepancies, almost the entire extraction space is used to address the crowding leaving very little margin of dental maneuvers. As Konstantonis et al. [23] showed, crowding is a major factor in the decision-making process when an orthodontist contemplates between extraction and non-extraction treatment. In this investigation, the borderline cases presented with similar amounts of crowding: − 2.51 and − 2.93 mm (*P* value = 0.448) of maxillary crowding and − 4.95 and − 5.37 mm (*P* value = 0.164) of mandibular crowding for the non-extraction and the extraction cases respectively (data not shown). The aforementioned amounts of crowding in the extraction cases allows the implementation of the desired biomechanics with regard to treatment goals in which vertical control plays a major role. Actually, more side effects are expected in borderline cases with a mild to moderate crowding rather than in clear-cut extraction cases with severe crowding.

Treatment duration varied significantly between the two groups in the present study. Extraction treatment lasted 2.79 years, whereas non-extraction treatment lasted 1.8 years. The four premolar extraction treatments lasted an average of 1 year or 55% longer than non-extraction treatments. Kim et al. [8] reported a mean treatment time of 2.3 years for extraction therapy with four first premolars; however, there was no control group treated without extractions. Longer treatment time for extraction therapies is also in concordance with the conclusions of Maveras et al. [33], but nevertheless, other factors like the implemented biomechanics, the operator’s experience, and patient’s compliance might have an additional impact on the treatment’s duration [34].

The main limitations of the present investigation are due to its retrospective nature. To overcome this, a large parent sample obtained from a previous study was used and a discriminant analysis was performed to reduce selection and susceptibility bias. A randomized control study would be ideal for such posttreatment comparisons, but randomization with regard to extractions is neither easy nor easily ethically justifiable. Furthermore, the study evaluated only class I extraction and non-extraction patients with a mean FMA of 28.37° (SD 3.96) and 26.85° (SD 5.86) respectively. Hence, no conclusion can be made about high-angle patients, although in these patients vertical control would be especially desirable. As in all cephalometric studies, landmark identification error is also a concern. Therefore, intra- and inter-examiner reliability was evaluated for both random and systematic errors. Still, since the majority of the patients were adolescents, different patterns of growth and variation between males and females should also be considered [35].

Since stability of the results achieved is a major goal for every orthodontist, long-term comparisons between the extraction and non-extraction treatment groups should also be considered. Extractions have an impact on the vertical skeletal dimension as shown in the present research study, but the possibility of relapse to the original dimension remains an issue to be clarified.

## Conclusions

Discriminant analysis was successful in identifying a group of morphologically similar patients, which were a borderline in regard to extractions. The choice of extraction or non-extraction treatment had an impact on the patients’ vertical skeletal dimensions. The overall difference between the two groups was significant with the extraction patients exhibiting a decrease in the vertical dimension when compared to the non-extraction patients. Patients treated with extractions of the four first premolars showed a slight decrease in the skeletal vertical measurements, whereas patients who received non-extraction treatment showed a slight increase. However, due to the small-scale intergroup differences, the results might be of little clinical significance. Still, treatment time was significantly higher in the extraction than in the non-extraction group. Further studies are needed to investigate the stability of the results achieved.
